# Evaluation of soil nutrients and berry quality characteristics of Cabernet Gernischet (*Vitis vinifera* L.) vineyards in the eastern foothills of the Helan Mountains, China

**DOI:** 10.3389/fpls.2024.1418197

**Published:** 2024-07-25

**Authors:** Yashan Li, Qi Li, Yinfang Yan, Weiqiang Liu, Chengdong Xu, Yanjun Wang, Lijun Nan, Xu Liu

**Affiliations:** ^1^ College of Enology, Northwest A&F University, Xianyang, China; ^2^ School of Resources, Environment and Chemistry, Chuxiong Normal University, Chuxiong, China; ^3^ College of Tobacco Science, Yunnan Agricultural University, Kunming, China

**Keywords:** Cabernet Gernischet, soil nutrient, berry quality, principal component analysis, cluster analysis

## Abstract

Soil is the basis of the existence of fruit tree and soil nutrients plays a crucial role in plant growth and berry quality. To investigate the characteristics and interrelationships between soil nutrients and berry quality in Cabernet Gernischet vineyards, this study focused on seven representative vineyards in the eastern foothills of the Helan Mountains. Fifteen soil physicochemical factors and 10 berry quality factors were measured, followed by variation analysis, correlation analysis, multiple linear regression (MLR), partial-least squares regression (PLSR), principal component analysis (PCA), and systematic cluster analysis. We identified the main soil nutrient indicators influencing berry quality and developed linear regression equations. Utilizing PCA, a comprehensive evaluation model for berry quality was constructed, which enabled the calculation and ranking of integrated berry quality scores. The results indicated that soil nutrients in the vineyards of the eastern foothills of the Helan Mountains are relatively deficient and alkaline. The coefficient of variation for soil nutrient factors ranged from 3.19 to 118.08% and for berry quality factors 2.41–26.37%. Correlation analysis revealed varying degrees of correlation between soil nutrient indicators and fruit quality indicators. PCA extracted four principal components with a cumulative contribution rate of 91.506%. Based on the scores of these components and their corresponding weights, a comprehensive model for evaluating the quality of Cabernet Gernischet berries was established. The vineyards were ranked from the highest to the lowest combined scores as Zhenbeibu (ZBB), Yuquanying (YQY), Dawukou (DWK), Beihaizi (BHZ), Shuxin (SX), Huangyangtan (HYT), and Hongde (HD). These findings provide insights into soil nutrient management and comprehensive quality assessment of vineyards in the eastern foothills of the Helan Mountains. In conclusion, this study offers a theoretical foundation for vineyard managers to enhance grape berries quality through soil nutrient management. This will aid in the diagnosis of vineyard soil nutrition and the efficient use of fertilizers, with critical practical and theoretical implications for the meticulous management of vineyards and the production of high-quality wines.

## 1 Introduction

The eastern foothills of the Helan Mountains in Ningxia, situated between 37°43′N – 39°23′N latitude, 105°45′E – 106°47′E longitude, and at an altitude of 1100 – 1120 m, form an open area flanked by the Helan Mountain alluvial fan and the Yellow River alluvial plain. This area spans 2410.7 km^2^, approximately 200 km in length from north to south and 5 – 30 km in width from east to west (Qi et al., 2019). This region experiences an arid climate within the temperate zone, as characterized by drought conditions, scant rainfall, abundant sunlight, significant day-night temperature variations, deep soil, and favorable conditions for irrigation from the Yellow River. Such distinctive geographical and natural attributes render it one of the premier ecological locales for wine grape cultivation in China (Wang et al., 2015). Cabernet Gernischt, a red wine grape variety renowned for its distinctive herbaceous flavor, thrives in the sandy soils of this region and exhibits drought tolerance, making it a prominent cultivated variety in the research region.

Soil serves as the foundation for fruit tree growth, and its nutrient status directly influences tree development, fruit yield, quality, and, ultimately, the economic profitability of orchards (Reeve et al., 2005). Extensive research conducted both domestically and internationally has investigated the relationship between vineyard soil nutrients and berry quality, revealing the pivotal role of soil nutrients in shaping and enhancing berry quality (Qi et al., 2019; Li Q. J. et al., 2024). However, many of these studies have primarily focused on simple correlation analyses between berry quality and soil nutrient factors, failing to adequately capture the complex relationship. Therefore, there is a need to delve deeper into this complex relationship through multivariate statistical analysis.

Partial least-squares regression (PLSR) emerges as a robust method for modeling multiple linear regression, which is capable of analyzing datasets with numerous, noisy, collinear, or incomplete variables across both dependent and independent variables (Wold et al., 2001). Thus, PLSR finds widespread application in analyzing the correlation between soil nutrients and berry quality (Zhang et al., 2018). Using the insights from such analyses, vineyard managers can identify the primary soil nutrient factors influencing berry quality, thereby facilitating improvements in the nutrient status and improving berry quality.

Various methods exist for assessing berry quality, including factor analysis (Feng et al., 2016), The technique for order preference by similarity to the ideal solution (TOPSIS) model (Hao et al., 2022), and principal component analysis (PCA) (Cao et al., 2014; Shi et al., 2015; Zheng et al., 2022), among others. As a commonly employed multidimensional data analysis method, PCA is widely applied in practical settings. In this study, seven representative vineyards served as the study sample. Fifteen soil nutrient indices and ten berry quality indices were assessed, followed by correlational analysis, PLSR, and MLR to elucidate the intrinsic relationship between soil nutrients and berry quality. In addition, PCA facilitated a comprehensive analysis and ranking of berry quality, offering valuable insights and guidance for local vineyard management.

## 2 Materials and methods

### 2.1 Experimental materials

**Figure 1:**
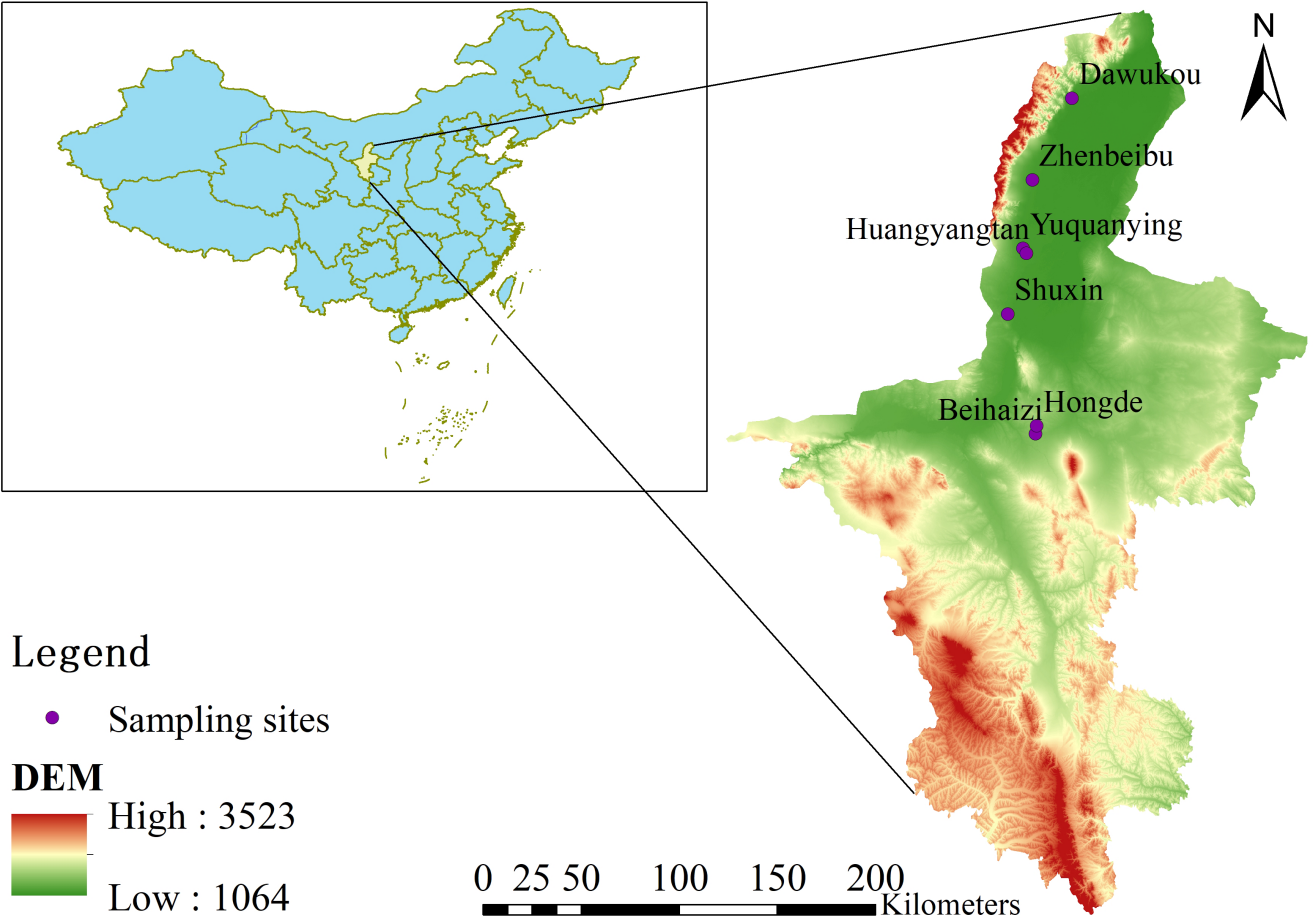
The location of the sampling sites.

We investigated the vineyards of Cabernet Gernischet situated in the wine-producing region of the eastern foothills of the Helan Mountains. Seven representative Cabernet Sauvignon vineyards were chosen from five sub-regions, including Shizuishan (Dawukou; DWK), Yinchuan (Zhenbeibu; ZBB), Yongning (Yuquanying [YQY] and Huangyangtan [HYT]), Qingtongxia (Shuxin; SX), and Hongsibu (Beihaizi [BHZ] and Hongde [HD]) (**Figure 1**). These vineyards are equipped with water and fertilizer systems for irrigation and fertilization. Drip irrigation is supplied via water sources from the Yellow River, which traverses the eastern part of the production area, as well as meltwater from the Helan Mountains. Fertilization practices aligned with the annual growth requirements of the grapes, whereas natural grass management models were implemented for field soil management. Grapevines were buried in the soil to protect them from severe winter cold. Refer to **Table 1** for detailed information on each vineyard.

**Table 1:** Basic information about Cabernet Gernischt vineyards from seven representative vineyards.

Sub-region	Vineyard	Longitude	Latitude	Altitude/(m)	Slope gradient/(°)	Row/within-row spaces/(m)
Shizuishan	Dawukou (DWK)	106°18′40″ E	38°59′14″ N	1074	2	1.50 × 3.20
Yinchuan	Zhenbeibu (ZBB)	106°4′21″ E	38°36′55″ N	1134	2	0.80 × 3.50
Yongning	Yuquanying (YQY)	106°1′24″ E	38°17′28″ N	1156	2	0.80 × 3.50
	Huangyangtan (HYT)	106°1′25″ E	38°17′19″ N	1162	3	0.80 × 3.50
Qingtongxia	Shuxin (SX)	105°55′12″ E	38°00′03″ N	1182	2	0.50 × 3.00
Hongsibu	Beihaizi (BHZ)	106°4′22″ E	37°27′33″ N	1368	3	0.80 × 4.00
	Hongde (HD)	106°4′49″ E	37°27′56″ N	1348	3	0.50 × 4.00

### 2.2 Sampling methods

Three soil sampling units measuring 10 m × 10 m were randomly selected from within each vineyard. Within each unit, an S-type soil sampling approach was employed, avoiding areas with fertilized ditches and drip irrigation zones. Fifteen soil sampling points were designated approximately 30–40 cm from the grape lines, and soil samples were collected using a tubular soil drill up to a depth of 40 cm from the soil surface. These samples were thoroughly mixed, and approximately 1 kg of soil was obtained by using the quartering method. Each soil sample was then placed into a self-sealing bag, labeled, and marked with the sampling site using a pencil, which served as the final soil sample for each unit. Three replicates were performed in each vineyard. All soil samples were subsequently placed in a freezer and transported to the laboratory. Approximately 100 g of fresh soil was selected from each sample for immediate identification of nitrate and ammonium nitrogen contents. The remaining soil samples were air-dried naturally in a shady and cool environment. Once dried, the soil samples were finely ground with a wooden stick and sifted through a sieve with a 1-mm aperture. After thorough mixing, two samples were selected using the quartering method for the determination of remaining soil chemical indicators.

Berry sampling was conducted during the commercial ripening period of the grapes within the same sampling area as the soil sampling. Fifty healthy clusters with consistent growth on both sides of the vine were randomly selected in a sampling unit. From each cluster, six grapes were harvested from different parts using miniature scissors. A total of 300 grape berries were collected from each sampling area and placed in individual self-sealing bags. Three bags were collected from each vineyard and transported to the laboratory freezer. From each bag, 100 grape berries were randomly selected for weighing, whereas another 100 berries were stored in a −80°C ultra-low temperature refrigerator for the measurement of the amount of anthocyanins, flavonols, and tannins. The remaining 100 grape berries were manually juiced, and the physicochemical factors of the grape juice were determined.

Mature grapes from the same sampling unit were hand-picked at the harvest date. Approximately 20 kg of healthy grapes per unit were obtained for small-scale wine production. The vinification was performed with reference to a previous method (Duan et al., 2022a). The winemaking was performed in triplicate for each vineyard.

### 2.3 Determination factors and methods

The total nitrogen (TN) content was determined according to the Kjeldahl method (Bao, 2000). Total phosphorus (TP) and available phosphorus (AP) contents were assessed by following the methods outlined by Bao (2000). Ammonium nitrogen (AN) and nitrate nitrogen (NN) were analyzed utilizing a continuous flow analyzer (AA3, SEAL Analytical GmbH, Germany) (Zhang et al., 2016). Total potassium (TK) was quantified through the NaOH fusion-atomic absorption method (Bao, 2000), whereas the available potassium (AK) content was measured using NH_4_OAc digestion-flame photometry (Bao, 2000). Organic matter (OM) content was determined using the potassium dichromate volumetric method with external heating (Zhang et al., 2020). The available iron (AFe), available zinc (AZn), available copper (ACu), and available manganese (AMn) in the soil were determined employing diethylenetriaminepentaacetic acid-triethanolamine extraction and flame/graphite furnace atomic absorption spectrometer (900T AAS, PerkinElmer Inc., USA) (Lindsay and Norvell, 1978). Exchangeable calcium and exchangeable magnesium were determined via atomic absorption spectrophotometry (PinAAcle 900F, PerkinElmer, America) (Bao, 2000). Soil pH was measured by using a pH meter (pHS-3C) in a soil: water (1:2.5) suspension (Zhang et al., 2021).

Berry weight was measured with an electronic balance (JY10002, Shanghai Hengping Instrument Co., Ltd, Shanghai, China). Longitudinal and transverse diameters were assessed with an electronic digital display vernier caliper (IP54, Shanghai Meinaite Industrial Co., LTD, Shanghai, China). Soluble solids of grape juice were measured using a handheld digital refractometer (PAL-1, Atago, Japan). Reducing sugar content was determined through Fehling’s solution-titration (Ma et al., 2017). Titratable acidity and grape juice pH were measured by using the Easyplus Titration System (Mettler Toledo, Columbus, OH, USA) and PHS-3C pH meter (Precision Science Instruments Co., Ltd., Shanghai, China), respectively. Tannins, anthocyanins, and flavonols were extracted and determined based on previous literature (Li Y. S. et al., 2024). Anthocyanins, flavonols and tannins are classified according to their substituents at the 3’ and 5’ positions of the B-ring of flavonoids, and the types of acylation at the 3’ position of the C-ring (Duan et al., 2022a). The basic physicochemical indicators of wine were determined by a Fourier-transformed infrared spectroscopy automatic wine analyzer (Lyza 5000 Wine, Anton Paar GMBH, Austria). The sensory evaluation of biological quality was conducted in a single day in a wine sensory analysis laboratory located in the College of Enology of Northwestern A&F University in Yangling, China. A 13-member panel of experienced professionals was appointed. The panelists were asked to taste the wines and perform descriptive analyses on each sample in a blind tasting. Each wine sample was scored on a 100-point scale based on a wine sensory rating scale.

### 2.4 Screening of main soil nutrient indices affecting grape berry quality and constructing regression equations

The PLSR method was primarily employed to screen soil nutrient factors, following the approach outlined by Wang et al. (2017). Using SPSS 25.0 software, the soil nutrient indices were designated as independent variables and each berry quality index was the dependent variable for PLSR analysis. Standardized regression equations were derived from the output results of the software by using the weight results of potential factors. Subsequently, based on the magnitude of standardized regression coefficients and professional expertise, the indices were screened. Multiple linear regression (input method) was then conducted using SPSS 25.0 to establish regression equations and to perform significance tests.

### 2.5 Statistical analyses

Original data were processed using Excel 2016 software (Microsoft, WA, America). One-way analysis of variance, PLSR, and multiple linear regression were conducted using SPSS 25.0 software (IBM, Chicago, IL, USA). Tukey’s multiple-range tests at a significance level of *P*< 0.05 were performed using SPSS 25.0. Origin 2023 software (OriginLab Corporation, Massachusetts, America) was utilized for correlation mapping and cluster analysis.

## 3 Results

### 3.1 Soil nutrients analysis of Cabernet Gernischet vineyards

#### 3.1.1 Soil nutrients profile

The statistical analysis results of soil nutrients in Cabernet Gernischet vineyards are summarized in **Table 2**. Macroelement analysis revealed significant disparities among vineyards. In the DWK vineyard, total nitrogen, total potassium, total phosphorus, and nitrate nitrogen levels were notably higher compared to those in other vineyards. YQY and HD vineyards exhibited significantly higher ammonium nitrogen levels, whereas ZBB demonstrated significantly elevated available potassium levels. Exchangeable magnesium levels of ZBB were significantly higher than for the other vineyards. Trace element distribution also showcased disparities, with DWK showing significantly higher available iron levels than other vineyards, and ZBB exhibiting notably higher available manganese levels. Conversely, the HD vineyard displayed significantly lower available copper and zinc contents when compared to the other vineyards. In addition, the organic matter content of different vineyards varied significantly. DWK had the highest organic matter content, that is, significantly higher than that for the other vineyards and 11.61-times greater than HD. Across all vineyards, soil exhibited alkalinity, with pH levels reaching 8.54 in the HYT vineyard, which was significantly higher than for the other vineyards. The DWK vineyard exhibited relatively favorable soil nutrient content, whereas SX, BHZ, and HD vineyards displayed comparatively impoverished soil nutrient content. When compared with the grading standard of soil nutrients in vineyards (Wang, 2016), the vineyard soil in the eastern foothills of the Helan Mountains was relatively poor, with low availability of most of the soil nutrient elements. The elevated pH may be one of the main factors limiting soil nutrient activation and nutrient retention in this region.

**Table 2:** Soil nutrient content across different vineyards.

Vineyard	TN/(g·kg^-1^)	TP/(g·kg^-1^)	TK/(g·kg^-1^)	OM/(g·kg^-1^)	NN/(mg·kg^-1^)	AN/(mg·kg^-1^)	AP/(mg·kg^-1^)	AK/(mg·kg^-1^)	ACu/(mg·kg^-1^)	AZn/(mg·kg^-1^)	AFe/(mg·kg^-1^)	AMn/(mg·kg^-1^)	ECa/(mg kg^-1^)	EMg/(mg kg^-1^)	Soil pH
DWK	1.46 ± 0.04a	0.68 ± 0.01a	19.11 ± 0.23a	45.26 ± 2.87a	75.25 ± 2.05a	1.67 ± 0.04d	9.65 ± 1.44ab	133.34 ± 0.49d	0.87 ± 0.03d	1.89 ± 0.06b	11.15 ± 0.32a	13.71 ± 0.15b	60.71 ± 3.54a	92.51 ± 0.73b	8.05 ± 0.02d
ZBB	0.90 ± 0.02b	0.50 ± 0.00c	15.85 ± 0.33bc	11.17 ± 0.25b	27.99 ± 1.15d	2.99 ± 0.18b	10.08 ± 0.35a	337.49 ± 0.26a	1.59 ± 0.00b	1.77 ± 0.04bc	6.15 ± 0.02b	20.78 ± 0.54a	56.50 ± 0.08ab	93.92 ± 0.14a	8.04 ± 0.01d
YQY	0.61 ± 0.03c	0.55 ± 0.01b	16.19 ± 0.11b	7.39 ± 0.12bc	46.00 ± 2.83c	3.76 ± 0.15a	7.49 ± 0.26ab	137.21 ± 3.21d	1.16 ± 0.02c	3.23 ± 0.07a	4.76 ± 0.10c	11.95 ± 0.23c	54.78 ± 0.24b	90.48 ± 0.01c	8.13 ± 0.03c
HYT	0.46 ± 0.01d	0.57 ± 0.06b	15.11 ± 0.25c	6.98 ± 0.23bc	9.40 ± 0.26e	1.77 ± 0.05d	7.33 ± 0.89b	102.49 ± 0.12f	3.84 ± 0.07a	1.04 ± 0.08d	4.32 ± 0.10d	7.96 ± 0.11e	54.01 ± 0.43b	90.34 ± 0.12c	8.54 ± 0.01a
SX	0.63 ± 0.01c	0.31 ± 0.00e	16.30 ± 0.21b	4.48 ± 2.05c	2.97 ± 1.38f	2.29 ± 0.28c	4.39 ± 0.13c	107.92 ± 0.00e	0.52 ± 0.01e	1.67 ± 0.08c	6.23 ± 0.06b	9.12 ± 0.08d	19.50 ± 0.13c	83.14 ± 0.00d	8.33 ± 0.01b
BHZ	0.59 ± 0.01c	0.47 ± 0.00d	16.65 ± 0.44b	7.30 ± 0.14bc	62.59 ± 2.94b	1.90 ± 0.05cd	7.57 ± 0.18ab	236.67 ± 0.54b	0.50 ± 0.00e	0.72 ± 0.01e	4.65 ± 0.02cd	8.85 ± 0.07d	58.72 ± 0.43ab	90.76 ± 0.16c	7.68 ± 0.04e
HD	0.33 ± 0.01e	0.55 ± 0.01b	16.16 ± 0.09b	3.90 ± 0.05c	42.33 ± 2.01c	3.95 ± 0.25a	8.18 ± 1.24ab	145.84 ± 2.05c	0.25 ± 0.00f	0.57 ± 0.01f	3.59 ± 0.04e	6.67 ± 0.04f	55.44 ± 0.81b	89.92 ± 0.09c	8.38 ± 0.04b

TN, TK, TP, OM, NN, AN, AP, AK, ACu, AZn, AFe, AMn, ECa, and EMg are abbreviations for total nitrogen, total potassium, total phosphorus, organic matter, nitrate nitrogen, ammonium nitrogen, available phosphorus, available potassium, available copper, available zinc, available iron, available manganese, exchangeable calcium, and exchangeable magnesium. Different small letters in the same column mean a significant difference at P< 0.05 among vineyards according to Tukey’s test. The same as below.

The variation of soil nutrient factors across Cabernet Gernischet vineyards is depicted in **Table 3**. Variations in different soil nutrient factors were found to vary significantly, with organic matter and nitrate exhibiting coefficients of variation > 100% across different vineyards, indicating a strong variability intensity. In contrast, total potassium, exchangeable magnesium, and soil pH have lowest coefficients of variation (< 10%), indicating weak variability. The residual soil nutrient index showed a coefficient of variation of 10% – 100%, indicating a middle range of intensity.

**Table 3:** Variations of soil nutrient indexes across seven vineyards of Cabernet Gernischet.

Soil nutrient index	Range	Mean ± SD	CV/(%)
Total nitrogen/(g·kg^-1^)	0.33–1.46	0.71 ± 0.35	49.30
Total phosphorus/(g·kg^-1^)	0.31–0.68	0.52 ± 0.11	21.15
Total potassium/(g·kg^-1^)	15.11–19.11	16.48 ± 1.16	7.04
Organic matter/(g·kg^-1^)	3.90–45.26	12.35 ± 13.61	110.20
Nitrate nitrogen/(mg·kg^-1^)	2.97–75.25	21.10 ± 26.52	125.69
Ammonium nitrogen/(mg·kg^-1^)	1.67–3.95	2.62 ± 0.88	33.59
Available phosphorus/(mg·kg^-1^)	4.39–10.08	7.81 ± 1.72	22.02
Available potassium/(mg·kg^-1^)	102.49–337.49	171.57 ± 79.19	46.16
Available copper/(mg·kg^-1^)	0.25–3.84	1.25 ± 1.14	91.20
Available zinc/(mg·kg^-1^)	0.57–3.23	1.56 ± 0.84	53.85
Available iron/(mg·kg^-1^)	3.59–11.15	5.84 ± 2.34	40.07
Available Manganese/(mg·kg^-1^)	6.67–20.78	11.29 ± 4.46	39.50
Exchangeable calcium/(mg kg^-1^)	19.50–60.71	51.38 ± 13.19	25.67
Exchangeable magnesium/(mg kg^-1^)	83.14–93.92	90.15 ± 3.15	3.49
Soil pH	7.68–8.54	8.16 ± 0.26	3.19

SD stands for standard deviation, CV means coefficient of variation. The same as below.

#### 3.1.2 Correlation of soil nutrient indices

**Figure 2:**
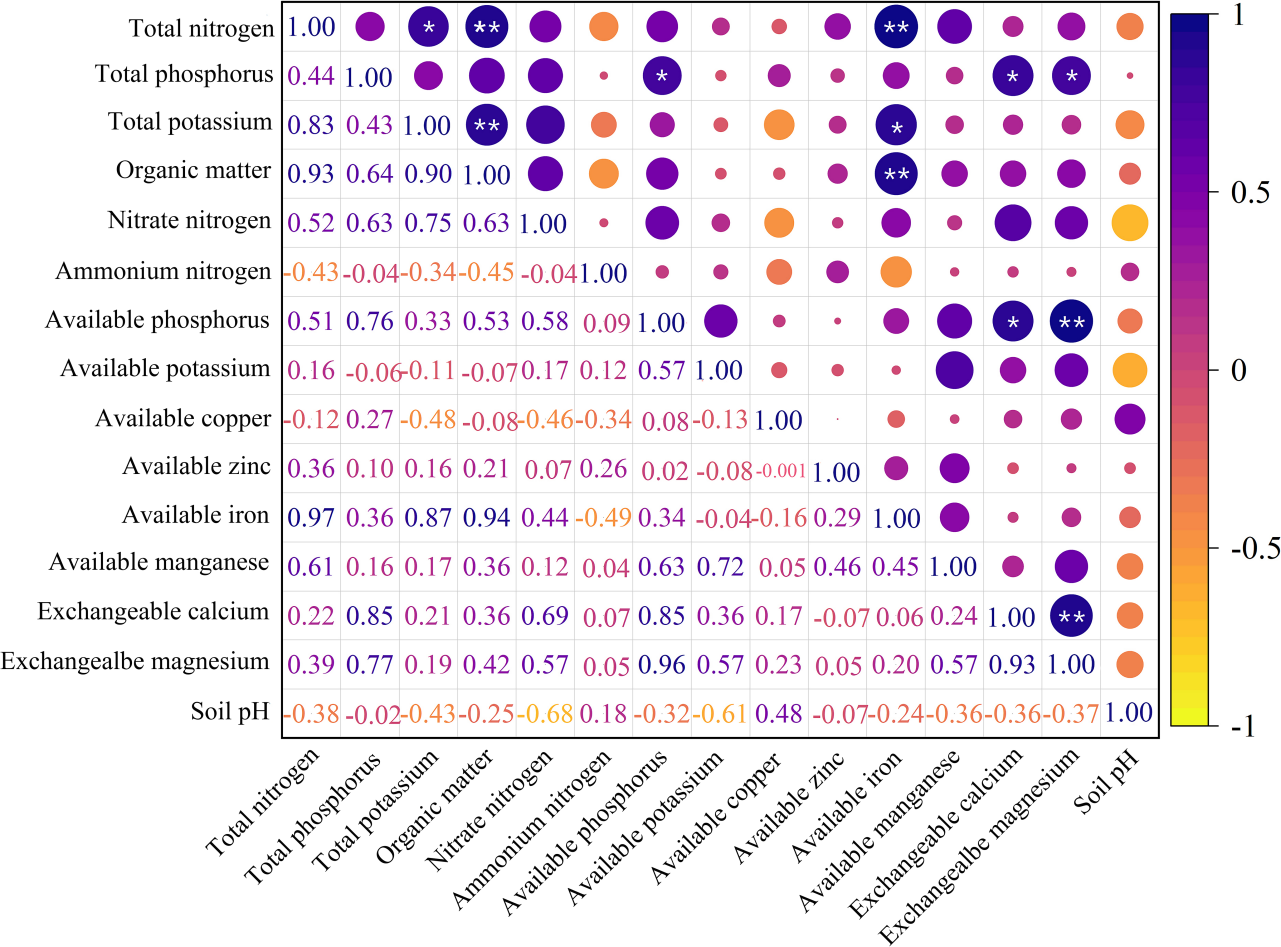
Correlation of the soil nutrient indices. * and ** indicated that the significance level reached *P*< 0.05 and *P*< 0.01, respectively.

The correlations among soil nutrients in the Cabernet Gernischet vineyards are depicted in **Figure 2**. The correlations between total nitrogen and total potassium, organic matter and available iron, respectively, were at a significant level, and, for the latter two, it was an extremely significant level. There was a significant correlation between total phosphorus and available phosphorus, exchangeable calcium, and exchangeable magnesium. Total potassium level was significantly correlated with the organic matter and available iron. There was a significant correlation between organic matter and available iron. The correlation between available phosphorus and exchangeable calcium and exchangeable magnesium was at a significant and extremely significant level, respectively. The correlation between exchangeable calcium and exchangeable magnesium also reached a highly significant level. All correlations among the above mentioned nutrients were positively correlated. The correlations between other soil nutrients did not reach significant levels. Overall, soil nutrient indicators were mainly positively correlated, with the synergistic effects being greater than the antagonistic ones.

### 3.2 Berry quality analysis of Cabernet Gernischet

#### 3.2.1 Berry quality profile

The results of grape quality index determination for Cabernet Gernischet are presented in **Table 4**. ZBB showed significantly higher berry weights and transverse diameters than did the other vineyards. HYT, SX, and BHZ showed lower longitudinal and transverse diameters than those shown by the remaining vineyards. The contents of reducing sugars and soluble solids in HD were significantly higher than those in the other vineyards, the contents of soluble solids and reducing sugars in DWK were significantly lower than those in the other vineyards, and the content of titratable acids in YQY was significantly lower than those in the other vineyards. Grape juice from berries in YQY showed the highest pH and that from berries in HD showed the lowest pH. In terms of the flavonoid content, ZBB and SX showed significantly higher tannin content than the other vineyards, while BHZ showed the highest anthocyanin and flavonol levels.

**Table 4:** Quality characteristics of grape berries of Cabernet Gernischet in seven vineyards.

Vineyard	Berry weight/g	Longitudinal diameter/mm	Transverse/mm	Soluble solid/(%)	Reducing sugar/(g·L^-1^)	Titratable acidity/(g·L^-1^)	Grape juice pH	Tannin(g·g^-1^)	Anthocyanin(g·g^-1^)	Flavonol/(g·g^-1^)
DWK	1.92 ± 0.08bc	14.19 ± 0.30ab	13.73 ± 0.26bc	21.20 ± 0.08e	189.80 ± 0.16f	4.53 ± 0.02c	3.85 ± 0.02b	22.15 ± 1.29d	61.64 ± 10.86a	4.80 ± 0.36c
ZBB	2.64 ± 0.14a	15.18 ± 0.54a	15.55 ± 0.31a	23.57 ± 0.12d	211.54 ± 0.21e	4.70 ± 0.01b	3.68 ± 0.01de	47.91 ± 1.21a	58.22 ± 0.98a	5.45 ± 0.01bc
YQY	2.23 ± 0.13b	15.07 ± 0.25a	14.42 ± 0.13b	25.47 ± 0.05b	230.68 ± 0.13b	3.35 ± 0.01f	3.90 ± 0.01a	31.75 ± 1.20bc	77.69 ± 1.11a	7.24 ± 0.07ab
HYT	1.48 ± 0.11d	12.74 ± 0.40cd	12.42 ± 0.21d	24.53 ± 0.12c	222.32 ± 0.15c	3.31 ± 0.00g	3.73 ± 0.01c	28.96 ± 1.08c	68.71 ± 11.02a	6.02 ± 0.77abc
SX	1.59 ± 0.18cd	12.87 ± 0.32cd	12.90 ± 0.55cd	23.23 ± 0.12d	213.84 ± 0.27d	3.60 ± 0.01e	3.66 ± 0.01ef	44.71 ± 2.61a	65.67 ± 2.33a	5.50 ± 0.38bc
BHZ	1.75 ± 0.12cd	12.45 ± 0.51d	12.29 ± 0.45d	24.43 ± 0.26c	221.61 ± 0.39c	4.75 ± 0.01a	3.70 ± 0.01cd	37.26 ± 1.53b	82.91 ± 4.59a	7.69 ± 0.42a
HD	1.42 ± 0.17d	13.80 ± 0.46bc	13.07 ± 0.32cd	27.00 ± 0.14a	245.30 ± 0.72a	4.06 ± 0.00d	3.63 ± 0.00f	25.43 ± 0.77cd	59.02 ± 3.73a	6.87 ± 0.23ab

Different small letters in the same column mean a significant difference at *P* < 0.05 among vineyards according to Tukey’s test.

**Figure 3:**
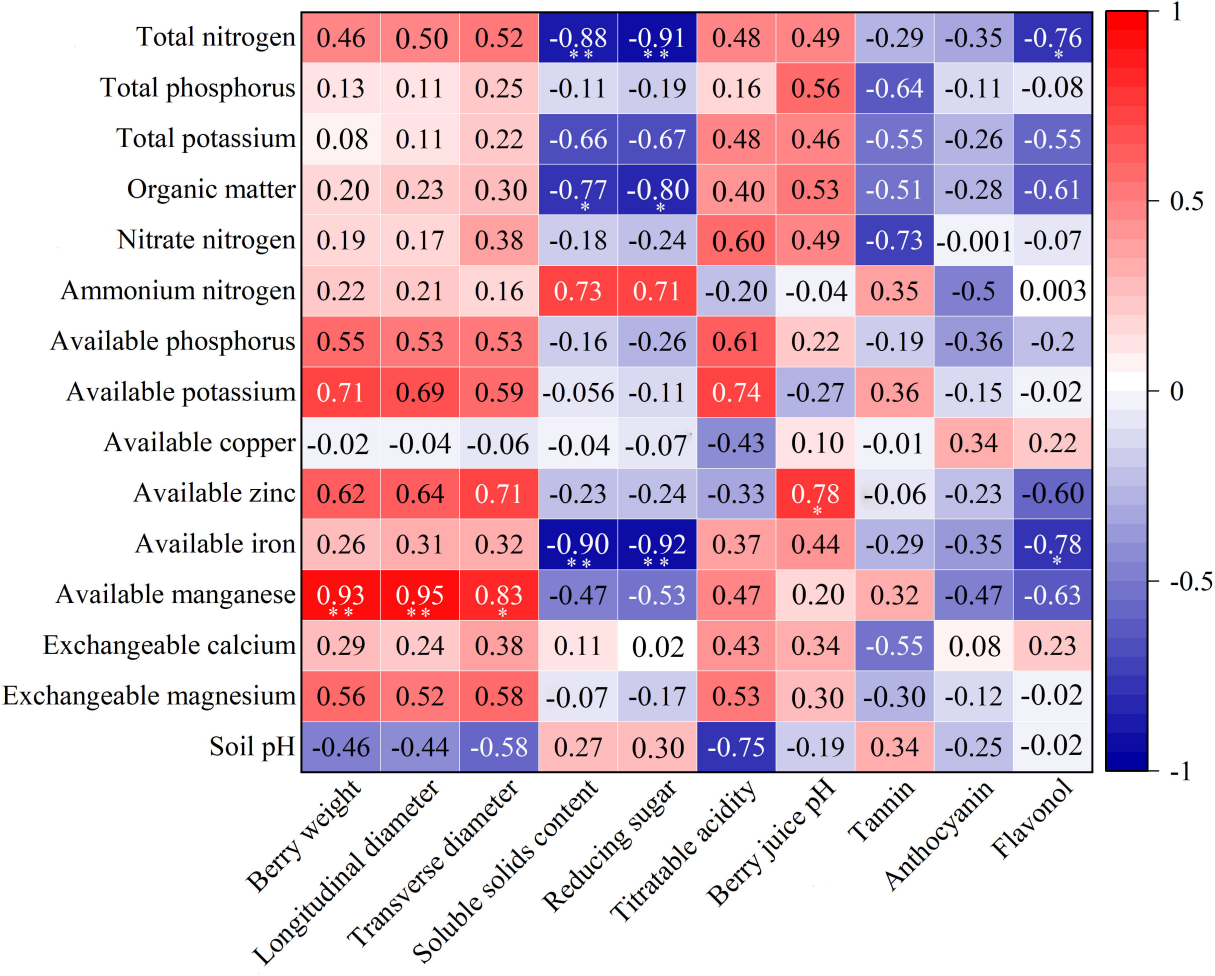
Correlation analysis of soil nutrients and berry quality indices. * and ** indicated that the significance level reached *P* < 0.05 and *P* < 0.01, respectively.

**Table 5** displays variations in the berry quality index for the seven vineyards. Different indicators varied widely among the vineyards, with higher degrees of variations observed in the tannin levels and berry weight, both of which were > 20% (**Figure 3**), followed by flavonoid levels, titratable acidity, and anthocyanin levels, all exhibiting variation coefficients > 10%. However, their coefficient of variation is< 100%, hence they belong to the medium level of variation. The coefficient of variation of longitudinal diameter, transverse diameter, soluble solid content, reduced sugar content, and berry juice pH was< 10%, which belonged to weak variation.

**Table 5:** Variations of berry quality indices among seven vineyards.

Berry quality index	Range	Mean ± SD	CV/(%)
Berry weight/g	1.42–2.64	1.86 ± 0.41	22.04
Longitudinal diameter/mm	12.45–15.18	13.76 ± 1.03	7.49
Transverse diameter/mm	12.29–15.55	13.48 ± 1.09	8.09
Soluble solid/%	21.20–27.00	24.20 ± 1.69	6.98
Reducing sugar/(g·L^-1^)	189.80–245.30	219.30 ± 15.95	7.27
Titratable acidity/(g·L^-1^)	3.31–4.75	4.04 ± 0.58	14.36
Berry juice pH	3.63–3.90	3.74 ± 0.09	2.41
Tannin/(g·L^-1^)	22.15–47.91	34.02 ± 8.97	26.37
Anthocyanin/(g·L^-1^)	58.22–82.91	67.69 ± 8.77	12.96
Flavonol/(g·L^-1^)	4.80–7.69	6.22 ± 0.98	15.76

#### 3.2.2 Flavonoid (anthocyanins, flavonols, and tannins) profiles of grape berries

The flavonoids in grape berries mainly contain three types of substances, anthocyanins, flavonols, and condensed tannins. In this study, 19 anthocyanins, nine flavonols, and 4 tannin units were detected. The content of these substances in grape berries across seven vineyards is presented in **Table 6**.

**Table 6:** Flavonoid components content of grape berries of Cabernet Gernischet across seven vineyards.

Flavonoid	Composition	DWK	ZBB	YQY	HYT	SX	BHZ	HD
Anthocy-anins^a^ (mg g^-1^)	Dp	7.08 ± 5.15a	2.26 ± 0.06a	3.51 ± 0.07a	2.03 ± 0.33a	5.30 ± 0.07a	4.34 ± 0.30a	4.98 ± 0.75a
	Cy	0.66 ± 0.10b	0.75 ± 0.04b	0.87 ± 0.01b	0.35 ± 0.04c	1.90 ± 0.11a	0.58 ± 0.04bc	0.82 ± 0.03b
	Pt	3.33 ± 0.54c	3.37 ± 0.11c	5.33 ± 0.05ab	3.34 ± 0.60c	6.39 ± 0.42a	5.66 ± 0.32a	3.88 ± 0.21bc
	Pn	4.41 ± 0.74ab	4.16 ± 0.07bc	5.50 ± 0.03ab	3.81 ± 0.65b	6.09 ± 0.04a	5.18 ± 0.32ab	4.94 ± 0.41ab
	Mv	23.56 ± 3.78b	24.71 ± 0.61ab	33.28 ± 0.50ab	28.73 ± 4.48ab	26.06 ± 1.99ab	36.20 ± 2.58a	23.32 ± 1.23b
	Dp-ac	0.80 ± 0.09c	1.04 ± 0.03bc	1.35 ± 0.07ab	0.76 ± 0.10c	1.72 ± 0.19a	1.23 ± 0.05bc	1.44 ± 0.15ab
	Cy-ac	1.06 ± 0.15a	0.30 ± 0.02b	1.04 ± 0.14a	0.76 ± 0.18ab	0.53 ± 0.07b	0.82 ± 0.04ab	0.35 ± 0.08b
	Pt-ac	0.9 ± 0.13c	0.96 ± 0.01c	1.41 ± 0.07abc	1.03 ± 0.11bc	1.56 ± 0.19ab	1.20 ± 0.06abc	1.60 ± 0.17a
	Pn-ac	0.26 ± 0.01bc	0.30 ± 0.02ab	0.37 ± 0.03a	0.29 ± 0.03ab	0.28 ± 0.02bc	0.34 ± 0.01ab	0.20 ± 0.02c
	Mv-ac	8.01 ± 1.19a	8.30 ± 0.22a	10.73 ± 0.22a	11.80 ± 1.77a	7.66 ± 0.75a	10.76 ± 0.79a	10.81 ± 0.60a
	Pt-caf	1.08 ± 0.12a	1.16 ± 0.03a	1.49 ± 0.03a	1.20 ± 0.20a	1.22 ± 0.50a	1.51 ± 0.10a	1.53 ± 0.23a
	Pn-caf	1.35 ± 0.11bc	1.73 ± 0.09ab	2.19 ± 0.11a	2.25 ± 0.34a	0.98 ± 0.14cd	1.87 ± 0.13ab	0.60 ± 0.03d
	Mv-caf	0.28 ± 0.02bcd	0.35 ± 0.01bc	0.31 ± 0.01bcd	0.36 ± 0.04b	0.26 ± 0.02cd	0.46 ± 0.01a	0.23 ± 0.01d
	cDp	0.30 ± 0.02bc	0.31 ± 0.01b	0.43 ± 0.01a	0.28 ± 0.03bc	0.27 ± 0.00bc	0.28 ± 0.02b	0.22 ± 0.01c
	cPn	0.17 ± 0.01bc	0.17 ± 0.01bc	0.17 ± 0.00bc	0.14 ± 0.00c	0.22 ± 0.02a	0.18 ± 0.01b	0.19 ± 0.01ab
	cMv	0.14 ± 0.00c	0.16 ± 0.00c	0.15 ± 0.00c	0.18 ± 0.01b	0.18 ± 0.00b	0.21 ± 0.01a	0.15 ± 0.01c
	tCy	0.67 ± 0.07bc	0.61 ± 0.01bc	0.85 ± 0.01ab	0.62 ± 0.10bc	0.56 ± 0.10bc	1.05 ± 0.08a	0.38 ± 0.01c
	tPt	0.88 ± 0.11a	0.83 ± 0.01a	1.08 ± 0.03a	0.87 ± 0.17ab	0.76 ± 0.02a	1.03 ± 0.07a	0.72 ± 0.03b
	tMv	6.69 ± 0.83ab	6.76 ± 0.10ab	7.64 ± 0.10ab	9.90 ± 1.89a	3.72 ± 0.94bc	10.01 ± 0.65a	2.66 ± 0.11c
Flavonols^b^ (mg g^-1^)	My-glc	1.09 ± 0.16b	1.06 ± 0.02b	1.36 ± 0.04ab	1.26 ± 0.16ab	1.23 ± 0.06b	1.68 ± 0.07a	1.01 ± 0.09b
	Qu-glcU	0.50 ± 0.06c	0.79 ± 0.04bc	1.38 ± 0.02ab	0.68 ± 0.16c	0.88 ± 0.16bc	1.50 ± 0.30a	1.58 ± 0.06a
	Qu-glc	0.67 ± 0.07c	1.11 ± 0.03bc	1.62 ± 0.02ab	1.11 ± 0.17bc	1.08 ± 0.22bc	1.46 ± 0.09ab	1.66 ± 0.06a
	La-gal	0.47 ± 0.03abc	0.41 ± 0bcd	0.52 ± 0.01ab	0.51 ± 0.05ab	0.36 ± 0.01d	0.55 ± 0.02a	0.40 ± 0.01cd
	Ka-glc	0.38 ± 0.02b	0.43 ± 0.03b	0.43 ± 0.00b	0.42 ± 0.03b	0.40 ± 0.04b	0.61 ± 0.02a	0.48 ± 0.01b
	La	0.33 ± 0.01b	0.33 ± 0.00b	0.38 ± 0.00b	0.47 ± 0.04ab	0.37 ± 0.06b	0.45 ± 0.02ab	0.57 ± 0.01a
	Ka-caf	0.52 ± 0.41ab	0.45 ± 0.40bc	0.58 ± 0.46a	0.60 ± 0.49a	0.35 ± 0.41c	0.51 ± 0.47ab	0.39 ± 0.47c
	Isorh-glc	0.41 ± 0.02a	0.40 ± 0.01a	0.46 ± 0.02a	0.49 ± 0.04a	0.41 ± 0.01a	0.47 ± 0.01a	0.47 ± 0.01a
	Sy-gal	0.42 ± 0.02ab	0.45 ± 0.01a	0.51 ± 0.01a	0.47 ± 0.05a	0.41 ± 0.03ab	0.46 ± 0.02a	0.33 ± 0.01b
Tannins^c^ (mg g^-1^)	EGC unit	14.40 ± 1.36bc	17.29 ± 0.06a	16.25 ± 0.19ab	14.30 ± 0.11bc	13.21 ± 0.15c	16.55 ± 0.1ab	15.18 ± 0.46abc
	C unit	7.01 ± 0.33ab	5.51 ± 0.09b	5.71 ± 0.46ab	5.71 ± 0.09ab	7.24 ± 0.36a	5.76 ± 0.14ab	6.05 ± 1.06ab
	EC unit	59.10 ± 3.86b	68.56 ± 0.69a	69.11 ± 0.46a	64.57 ± 0.43a	69.79 ± 0.58a	64.86 ± 0.73a	64.98 ± 0.91a
	ECG unit	19.49 ± 1.62a	8.64 ± 0.81e	10.88 ± 0.45cde	13.47 ± 0.64bc	9.76 ± 0.54de	14.20 ± 1.47b	12.42 ± 0.64bcd

^a^Expressed as malvidin-3-O-glucoside equivalents. ^b^Expressed as quercetin-3-O-glucoside equivalents. ^c^Expressed as epicatechin equivalents. Different small letters in the same line mean a significant difference at P< 0.05 among vineyards according to Tukey’s test. Dp, Delphinidin-3-O-glucoside; Cy, Cyanidin-3-O-glucoside; Pt, Petunidin-3-O-glucoside; Pn, Peonidin-3-O-glucoside; Mv, Malvidin-3-O-glucoside; Dp-ac, Delphinidin-3-O-acetylglucoside; Cy-ac, Cyanidin-3-O-acetylglucoside; Pt-ac, Petunidin-3-O-acetylglucoside; Pn-ac, Peonidin-3-O-acetylglucoside; Pt-caf, Petunidin-(6-O-caffeoyl) glucoside; Mv-ac, Malvidin-3-O-acetylglucoside; cDp, Delphinidin-3-(6-O-coumaroyl) glucoside (cis isomer); Pn-caf, Peonidin-(6-O-caffeoyl) glucoside; cPn, Peonidin-3-(6-O-coumaroyl) glucoside (cis isomer); cMv, Malvidin-3-(6-O-coumaroyl) glucoside (cis isomer); Mv-caf, Malvidin-(6-O-caffeoyl) glucoside; tCy, Cyanidin-(6-O-coumaryoyl) glucoside (trans isomer); tPt, Petunidin-(6-O-coumaryoyl) glucoside (trans isomer); tMv, Malvidin-3-(6-O-coumaroyl) glucoside (trans isomer); My-glc, Myricetin-glucoside; Qu-glcU, Quercetin-glucuronide; Qu-glc, Quercetin-glucoside; La-gal, Laricitrin-3-O-galactoside; Ka-glc, Kaempferol-glucoside; La, Laricitrin-3-O-rhamnose-7-O-trihydroxycinnamic; Ka-caff, Kaempferol-3-O-caffeoylate; Isorh-glc, Isorhamnetin-3-O-glucoside; Sy-gal, Syringetin-3-O-galactoside; EGC, (-)-epigallocatechin; C, (+)-catechin; EC, (-)-epicatechin; ECG, (-)-epicatechin-3-O-gallate. The same as below.

Among the 19 anthocyanin monomers, malvidin-3-O-glucoside (Mv) has the relatively highest content. There were significant differences in the different anthocyanin monomers among the seven vineyards, except for delphinidin-3-O-glucoside (Dp), Malvidin-3-O-acetylglucoside (Mv-ac), and petunidin-(6-O-caffeoyl) glucoside (Pt-caf). The delphinidin-3-(6-O-coumaroyl) glucoside (cis isomer) (cDp) of YQY, cyanidin-3-O-glucoside (Cy) of SX, malvidin-3-(6-O-coumaroyl) glucoside (cis isomer) (cMv) and malvidin-(6-O-caffeoyl) glucoside (Mv-caf) of BHZ were significantly higher than that of other vineyards.

Of the nine flavonol monomers, myricetin-glucoside (My-glc), quercetin-glucuronide (Qu-glcU), and quercetin-glucoside (Qu-glc) had higher levels. Kaempferol-glucoside (Ka-glc) in BHZ was significantly higher than that in the other vineyards. There was no significant variation in Isorhamnetin-3-O-glucoside (Isorh-glc) content across different vineyards. The (-)-epicatechin (EC unit) showed the relatively highest content of the four monomers of tannins, but it was significantly lower in DWK than in the other vineyards. Conversely, the (-)-epicatechin-3-O-gallate (ECG unit) in DWK was significantly higher than the others.

#### 3.2.3 Correlation of berry quality indicators

**Figure 4:**
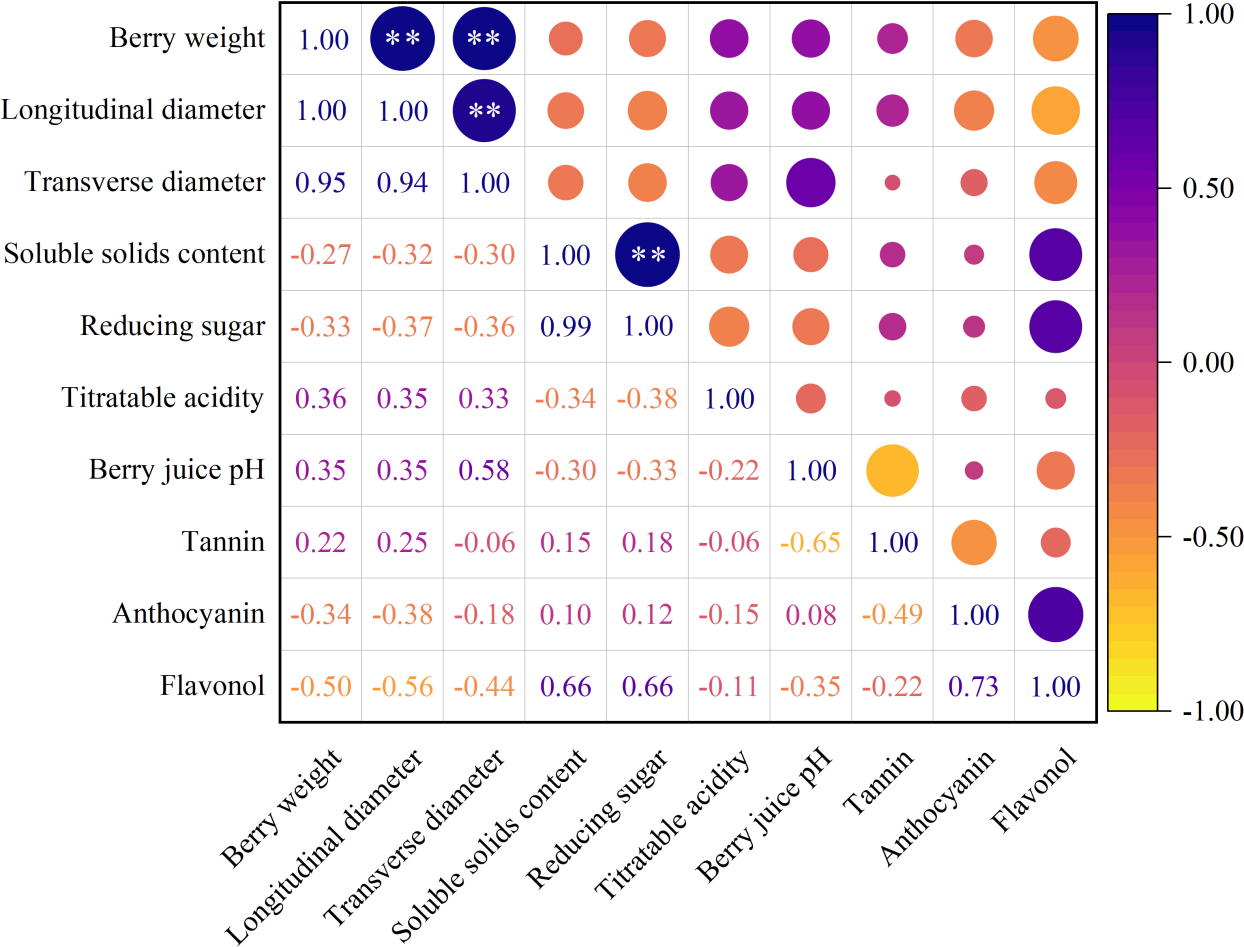
Correlation of berry quality indices. ** indicated that the significance level reached *P* < 0.01.

The results of the correlation analysis of berry quality indicators are presented in **Figure 4**. The berry weight was significantly positively correlated with the longitudinal and transverse diameters. The longitudinal diameter was significantly positively correlated with the transverse diameter. In addition, soluble solids and reduced sugar contents with correlation coefficients >0.90 were significantly positively correlated with each other.

#### 3.2.4 Assessment of the enological quality of each berry sample

The berry weight and volume of DWK were moderate. Moderate to strong acidity was attributable to northerly location, lack of heat, fewer soluble solids and reduced sugar content. There was also minor flavonoid, especially tannin, content. These properties make DWK’s grape berries suitable for the production of low-alcohol fresh wines. The vineyards of ZBB and YQY are located in the heart of the wine-producing region at the eastern foothills of the Helan Mountains, close to the mountain’s abundant sources of heat. Not only are the berries large and heavy, but they also contain more amount of soluble solids and reducing sugars, have appropriate acidity, tannins, anthocyanins, flavonols and other flavoring substances, and have the potential to make superior-quality wines. Although the vineyard is rich in calories, the grape berries of HYT obtain higher soluble solids and reduced sugars, and the resulting wine has a higher alcohol content. However, it has a relatively low acid content and tannin content, which easily creates an imbalance in the balance triangle of alcohol, acid, and tannin. SX and BHZ berries are medium-sized, moderately soluble solids, low in sugar and tannins, and have a degree of acidity to produce balanced wines. HD’s grape berries are rich in sugar, and the resulting wines are elevated in alcohol content. However, the lack of anthocyanins and tannins can produce wines that are dull and unbalanced in flavor.

### 3.3 Correlational analysis of soil nutrient and berry quality indicators

To determine the correlation between soil nutrient indicators and the berry quality indicators, a correlation analysis was performed (**Figure 3**). Berry weight, longitudinal diameter, and transverse diameter were positively correlated with all soil nutrient indicators, except for pH, and the correlation with available manganese reached significant or extremely significant levels. Soluble solids and reduced sugar displayed a high degree of agreement in relation to soil nutrient indicators. They were only positively correlated with ammonium nitrogen, exchangeable calcium, and soil pH, but negatively correlated with other soil nutrient indicators. The relationship between soluble solids and total nitrogen, organic matter, and available iron was significant or highly significant. The same was true for reducing sugar. Flavonol was negatively correlated with most of the soil nutrient indicators and significantly negatively correlated with total nitrogen and available iron. In addition, there was a significant positive correlation between the berry juice pH and available zinc. None of the other interactions reached significant levels.

### 3.4 Correlation analysis of soil nutrient and grape berry flavonoids

The results of the correlation analysis between soil nutrient indicators and grape berry flavonoids are shown in **Figure 3**. Among the anthocyanin monomers, cyanidin-3-O-glucoside (Cy) and TP, Cy and ECa, Cy and EMg, petunidin-3-O-glucoside (Pt) and TP, Pt and AP, peonidin-3-O-glucoside (Pn) and TP, delphinidin-3-(6-O-coumaroyl) glucoside (cis isomer) (cDp) and ACu, and cyanidin-(6-O-coumaryoyl) glucoside (trans isomer) (tCy) and soil pH were significantly negatively correlated, while the correlation between Cy and ECa reached a highly significant level. Delphinidin-3-O-glucoside (Dp) was positively correlated with TK, peonidin-(6-O-caffeoyl) glucoside (Pn-caf), and AZn, with the latter reaching extremely significant levels of correlation. Among the flavonoids monomers, quercetin-glucoside (Qu-glc) was negatively correlated with TN and AFe and laricitrin-3-O-rhamnose-7-O-trihydroxycinnamic (La) with TN and AMn, respectively. In tannin monomers, the EC unit was negatively correlated with OM, while the ECG unit was positively correlated with TK and OM, respectively. The correlation between other flavonoids monomer components and soil nutrient index was not significant.

### 3.5 Screening of main soil nutrient factors affecting berry quality of Cabernet Gernischet and the establishment of regression equations

Soil nutrient and berry quality indices are two different normal distribution aggregates. While soil nutrients affect berry quality, internal relationships between the two are complex. Twelve terms exhibited highly significant correlations among soil nutrient indicators, with correlation coefficients > 0.75 (**Figure 2**), thereby indicating multiple collinearities among the indicators. Thus, a simple linear analysis cannot reveal the true relationship between soil nutrients and berry quality. Hence, PLSR analysis was performed by considering the vineyard soil nutrient indicators as an independent variable and the berry quality indicators as a dependent variable to determine the multiple-linear relationship between soil nutrients and berry quality (Wold et al., 2001). The variable selection method in PLSR was conducted in accordance with the regression coefficients (Mehmood et al., 2012). Additionally, considering screened soil nutrient indicators as an independent variable and the corresponding berry quality index as a dependent variable, MLR was performed sequentially to establish the linear regression equations of berry quality and soil nutrient indicators (**Table 7**). The regression coefficients and symbols of the equations could reflect the important degrees and positive or negative impacts of the effects of different soil nutrient indicators on the berry quality factors (Zhang et al., 2018). Furthermore, a significance test was conducted on the established regression equations. The results were significant (**Table 7**), which suggested that the constructed regression equations were reliable.

**Table 7:** Selection of soil nutrient indices and construction of regression equations affecting berry qualities.

Berry quality	Soil nutrient factors	Regression equation	*R* ^2^	P value
Berry weight/(g)	*X*_8_, *X*_10_, *X*_11_, *X*_12_	*Y*_1_ = 0.697 + 0.003 *X*_8_+0.246 *X*_10_-0.005 *X*_11_+0.033 *X*_12_	0.986	0.009
Longitudinal diameter/(mm)	*X*_8_, *X*_10_, *X*_12_	*Y*_2_ = 10.355 + 0.006 *X*_8_+0.627 *X*_10_+0.102 *X*_12_	0.998	<0.001
Transverse diameter/(mm)	*X*_10_, *X*_12_, *X*_15_	*Y*_3_ = 23.850 + 0.577 *X*_10_+0.110 *X*_12_-1.499 *X*_15_	0.899	0.019
Soluble solid/(%)	*X*_1_, *X*_6_, *X*_11_	*Y*_4_ = 24.921-1.778 *X*_1_+0.770 *X*_6_-0.252 *X*_11_	0.862	0.030
Reducing sugar/(g·L^-1^)	*X*_1_, *X*_6_, *X*_11_	*Y*_5_ = 226.018-30.963 *X*_1_+6.945 *X*_6_-0.497 *X*_11_	0.909	0.016
Titratable acidity/(g·L^-1^)	*X*_3_, *X*_8_, *X*_9_, *X*_10_	*Y*_6_ = - 1.420 + 0.299 *X*_3_+0.006 *X*_8_-0.026 *X*_9_-0.258 *X*_10_	0.992	0.005
Berry juice pH	*X*_5_, *X*_8_, *X*_9_, *X*_10_, *X*_15_	*Y*_7_ = 4.120 + 0.002 *X*_5_-0.0004 *X*_8_+0.033 *X*_9_+0.079 *X*_10_-0.068 *X*_15_	0.999	0.024
Tannin/(g·L^-1^)	*X*_5_, *X*_7_, *X*_9_, *X*_12_	*Y*_8_ = 32.796-0.530 *X*_5_+3.275 *X*_7_-5.800 *X*_9_+0.272 *X*_12_	0.978	0.015
Anthocyanin/(g·L^-1^)	*X*_6_, *X*_7_, *X*_9_, *X*_12_, *X*_15_	*Y*_9_ = 379.012 + 0.649 *X*_6_-1.864 *X*_7_+7.272 *X*_9_-1.323 *X*_12_-35.849 *X*_15_	0.999	0.017
Flavonol/(g·L^-1^)	*X*_1_, *X*_6_, *X*_10_, *X*_11_, *X*_12_	*Y*_10_ = 11.621 + 7.815 *X*_1_-0.308 *X*_6_-0.243 *X*_10_-1.332 *X*_11_-0.177 *X*_12_	0.999	<0.001

*Y*_1_-*Y*_10_ represent for berry weight, longitudinal diameter, transverse diameter, soluble solid, reducing sugar titratable acidity, berry juice pH, tannin, anthocyanin and flavonol, respectively; *X*_1_-*X*_15_ stand for total nitrogen, total phosphorus, total potassium, organic matter, nitrate nitrogen, ammonium nitrogen, available phosphorus, available potassium, available Cu, available Zn, available Fe, available Mn, exchangeable calcium, exchangeable magnesium and soil pH, respectively.

As shown in **Table 7**, each indicator of berry quality was affected mainly by the various nutrients (except for soluble solids and reducing sugar). For example, berry weight was primarily affected by available potassium, available zinc, available iron, and available manganese. Available potassium, available zinc and available manganese had positive effects on the berry weight, available zinc had the largest effect on the berry weight, and iron had a negative effect. Notably, soluble solids and reducing sugars were affected by total nitrogen, ammonium nitrogen and available iron. Total nitrogen and ammonium nitrogen had a negative effect on both, while ammonium nitrogen had a positive effect. Among them, total nitrogen had the greatest effect on both. This effect can be attributed to the high correlation between soluble solids and reduced sugar content (**Figure 4**).

### 3.6 PCA of berry quality indices

The berry quality indices of the seven vineyards were analyzed by performing PCA (**Tables 8**, **9**). Following the principle that the eigenvalues are > 1.0, four principal components were identified, and the cumulative contribution rate reached 91.506%. The first principal component accounted for 38.493% of the original information content, and berry weight and longitudinal and transverse diameters displayed high positive coefficients (0.794, 0.695, and 0.818, respectively). Soluble solids, reducing sugar, and flavonol contents showed high negative coefficients (−0.691, −0.730, and −0.707, respectively). The results indicated that the greater the first principal component, the higher the berry weight and longitudinal and transverse diameters and the lower the soluble solid, reducing sugar, and flavonoid contents. The second principal component accounted for 22.229% of the original information content, with high positive coefficients for berry weight, longitudinal and transverse diameters, and soluble solid, reduced sugar, and flavonol contents, with high loading values of 0.530, 0.644, 0.556, 0.639, 0.598, and 0.565, respectively. The third principal component accounted for 17.144% of the original information, and the loading value was 0.925. Tannin content had a larger negative coefficient, and the loading value was −0.612, indicating that the larger is the third principal component, the higher is the berry juice pH and the lower is the tannin content. The fourth principal component accounted for 13.650% of the original information, with large positive coefficients and loading values of 0.529 and 0.692 for tannin and anthocyanin contents, respectively, indicating that the greater the fourth principal component, the higher the is tannin and anthocyanin contents.

**Table 8:** Initial eigenvalues and contribution rates of principle.

Principal components	Initial eigenvalues	Contribution rates %	Cumulative contribution rates %
1	3.849	38.493	38.493
2	2.223	22.229	60.722
3	1.714	17.144	77.866
4	1.364	13.650	91.506
5	0.826	8.260	99.766
6	0.023	0.234	100.000
7	6.358E-16	6.358E-15	100.000
8	1.629E-16	1.629E-15	100.000
9	-1.723E-16	-1.723E-15	100.000
10	-2.933E-16	-2.933E-15	100.000

### 3.7 Comprehensive evaluation of the berry quality of Cabernet Gernischet

PCA is a dimensionality reduction algorithm that transforms multiple indicators into a small number of principal components. These components are linear combinations of the original variables and are uncorrelated with each other, capturing most of the information present in the original data (Salerno et al., 2013). Specific calculation procedures can be found in the work of Abdi and Williams (2010). PCA has been effectively utilized for the comprehensive assessment of fruit quality (Cao et al., 2014; Shi et al., 2015; Zheng et al., 2022). This method is also utilized to assess the overall quality of grape berry in this study. According to the evaluation method, the loading value of the principal components of each quality index (**Table 9**) was divided by the square root of the eigenvalue corresponding to each principal component (**Table 8**) to obtain the eigenvector corresponding to each quality index of the four principal components (Zheng et al., 2022; Wang et al., 2023). Furthermore, score formulae (Equations 1–4) for the four principal components were obtained using the eigenvectors as weights (Wang H. D. et al., 2021; Tian et al., 2024). As given below:

**Table 9:** Loading matrix of the principle components of fruit quality indexes.

Quality Index	Principal components			
	1	2	3	4
Berry weight/g	0.794	0.530	-0.011	0.278
Longitudinal diameter/mm	0.695	0.644	0.092	-0.285
Transverse/mm	0.818	0.556	-0.105	-0.105
Soluble solid/%	-0.691	0.639	-0.226	-0.234
Reducing sugar/(g·L^-1^)	-0.730	0.598	-0.244	-0.221
Titratable acidity/(g·L^-1^)	0.409	-0.136	-0.319	0.448
Berry juice pH	0.314	0.209	0.925	0.001
Tannin/(g·L^-1^)	0.263	0.165	-0.612	0.529
Anthocyanin/(g·L^-1^)	-0.472	0.209	0.493	0.692
Flavonol/(g·L^-1^)	-0.707	0.565	0.099	0.362


(1) $$F_{1}=0.405\;Z_{1}+0.354\;Z_{2}+0.417\;Z_{3}-0.352\;Z_{4}-0.372\;Z_{5}+0.208\;Z_{6}+0.160\;Z_{7}+0.134\;Z_{8}-0.241\;Z_{9}-0.360\;Z_{10}$$

(2) $$F_{2}=0.355\;Z_{1}+0.432\;Z_{2}+0.373\;Z_{3}+0.429\;Z_{4}+0.401\;Z_{5}-0.091\;Z_{6}+0.140\;Z_{7}+0.11\;Z_{8}+0.140\;Z_{9}+0.379\;Z_{10}$$

(3) $$F_{3}=-0.008\;Z_{1}+0.070\;Z_{2}-0.080\;Z_{3}-0.173\;Z_{4}-0.186\;Z_{5}-0.244\;Z_{6}+0.707\;Z_{7}-0.467\;Z_{8}+0.377\;Z_{9}+0.076\;Z_{10}$$

(4) $$F_{4}=0.238\;Z_{1}-0.244\;Z_{2}-0.090\;Z_{3}-0.200\;Z_{4}-0.189\;Z_{5}+0.384\;Z_{6}+0.001\;Z_{7}+0.453\;Z_{8}+0.593\;Z_{9}+0.310\;Z_{10}$$

where *F*_1_–*F*_4_ represent the fractions of the principal components of fruit quality indices for different vineyards, and *Z*_1_–*Z*_10_ represents ten quality indices, including berry weight, longitudinal diameter, transverse diameter, soluble solid, reducing sugar, titratable acidity, berry juice pH, tannin, anthocyanin, and flavonol contents.

Using the variance contribution rates corresponding to the four principal components as weight coefficients, an integrated evaluation model Equation for berry quality was developed from the weighted sum of the principal component scores and the corresponding weight coefficients (Nie et al., 2019; Zhao et al., 2023):


(5) $$F=0.421\;F_{1}+0.243\;F_{2}+0.187\;F_{3}+0.149\;F_{4}$$

The model was used to obtain comprehensive berry quality scores for the seven vineyards (**Table 10**). The higher the overall score, the better the overall berry quality (Wu et al., 2022). As shown in **Table 10**, the overall ranking of fruit quality from highest to lowest was as follows: ZBB, YQY, DWK, BHZ, SX, HYT, and HD.

**Table 10:** Principal component score and comprehensive evaluation ranking.

Location	Principal component score				Comprehensive sore	Ranking
	*F*_1_	*F*_2_	*F*_3_	*F*_4_		
DWK	2.308	-1.745	1.462	-0.483	0.749	3
ZBB	3.008	1.068	-1.697	0.335	1.259	1
YQY	0.003	2.478	1.768	-0.028	0.930	2
HYT	-1.460	-0.914	0.432	-0.650	-0.853	6
SX	-0.250	-1.302	-0.846	0.150	-0.557	5
BHZ	-1.695	-0.235	-0.003	2.230	-0.439	4
HD	-1.914	0.651	-1.117	-1.555	-1.088	7

In order to verify the reliability of the results of the comprehensive evaluation of grape berry quality, the wines from different vineyards were tested for physical and chemical indexes (**Supplementary Table 1**) and sensory evaluation (**Supplementary Table 2**). As shown in **Supplementary Table 2**, wines from YQY and ZBB were rated excellent by sensory ratings, wines from HD vineyards were rated acceptable by sensory ratings, and wines from other vineyards were rated good by sensory ratings. It can be seen that the results of the sensory assessment are consistent with the results of the integrated evaluation of the quality of the berries.

### 3.8 Cluster analysis

**Figure 5:**
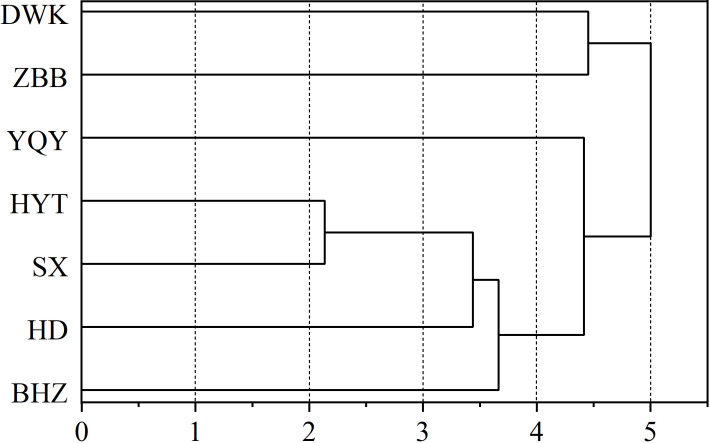
Cluster analysis of grape berry quality.

Cluster analysis was performed on the normalized berry quality indices (**Figure 5**). When the Euclidean distance was 4, the seven Cabernet Gernischet vineyards were divided into three categories. The first category comprised DWK and ZBB showing longer longitudinal and transverse diameters and lower soluble solid and reducing sugar contents. The second category comprised only YQY showing longer longitudinal diameter, higher berry juice pH, higher anthocyanin and flavonol content, and lower titratable acidity. The third category comprised the remaining vineyards displaying lower berry weights, shorter longitudinal and transverse diameters, and higher anthocyanin content. The results of the systematic clustering analysis were consistent with those of the integrated score ranking of PCA, which indicated the reliability and consistency of the present results.

## 4 Discussion

The unique terroir characteristics are the primary reason why the eastern foothills of the Helan Mountains wine region has become a world-renowned wine region (Yang et al., 2024). The study results revealed significant variations in soil nutrient levels among different vineyards within this wine region. It is well-established that diverse soil nutrient conditions can significantly impact the quality of grape berries. Therefore, this study’s findings offer valuable insights into how grape berry quality responds to soil nutrient properties in the eastern foothills of the Helan Mountains, and provide a solid foundation for vineyard soil management.

Soil properties play a significant role in influencing grape berry composition and the resulting wine quality (Cheng et al., 2014; Zerihun et al., 2015). Among all soil properties, soil nutrients are the most easily regulated and managed (Sun, 2022). It is crucial to investigate the relationship between soil nutrients and grape berry quality in order to provide guidance for vineyard production and management. The impact of nitrogen on grape berry quality primarily occurs through its direct influence on grape metabolism and indirect effects on vigor and yield (Poni et al., 2018). Numerous studies have indicated that high levels of nitrogen fertilization can decrease soluble solids content in berries while increasing titratable acidity content (Delgado et al., 2004; Thomidis et al., 2016). In this study, the correlation between different forms of nitrogen and fruit quality indexes was inconsistent (**Figure 3**), mainly due to the varying functions performed by different forms of nitrogen. Despite its significant role in plant growth and development, there is relatively limited literature regarding the effects of phosphorus on grape fruit quality. Amongst the few available experiments, only a minimal effect of phosphorus on grape berry quality has been observed (Topalović et al., 2011; Schreiner et al., 2013; Brunetto et al., 2015). The results of this study also align with these findings as evidenced by small correlation coefficients between both total and available phosphorus and grape berry quality indicators (**Figure 3**). However, Duan et al. (2022b) demonstrated a positive correlation between available phosphorus and the content of reducing sugars and anthocyanins in grape berries. However, the results of this study are contradictory, which may be attributed to differences in species and regions. It is well known that grapevines require a large amount of potassium, which plays an essential role in sugar accumulation in berries (Brunetto et al., 2015). In most cases, potassium is positively correlated with soluble solids (Ramos and Romero, 2017; Tassinari et al., 2022). Although the results of this study contradict this general trend, it is worth noting that the correlation coefficient is small, similar to the findings of Ciotta et al. (2021). This discrepancy may be due to the abundance of total and available potassium in all vineyards according to vineyard soil nutrient grading standards (Wang, 2016).

**Figure 6:**
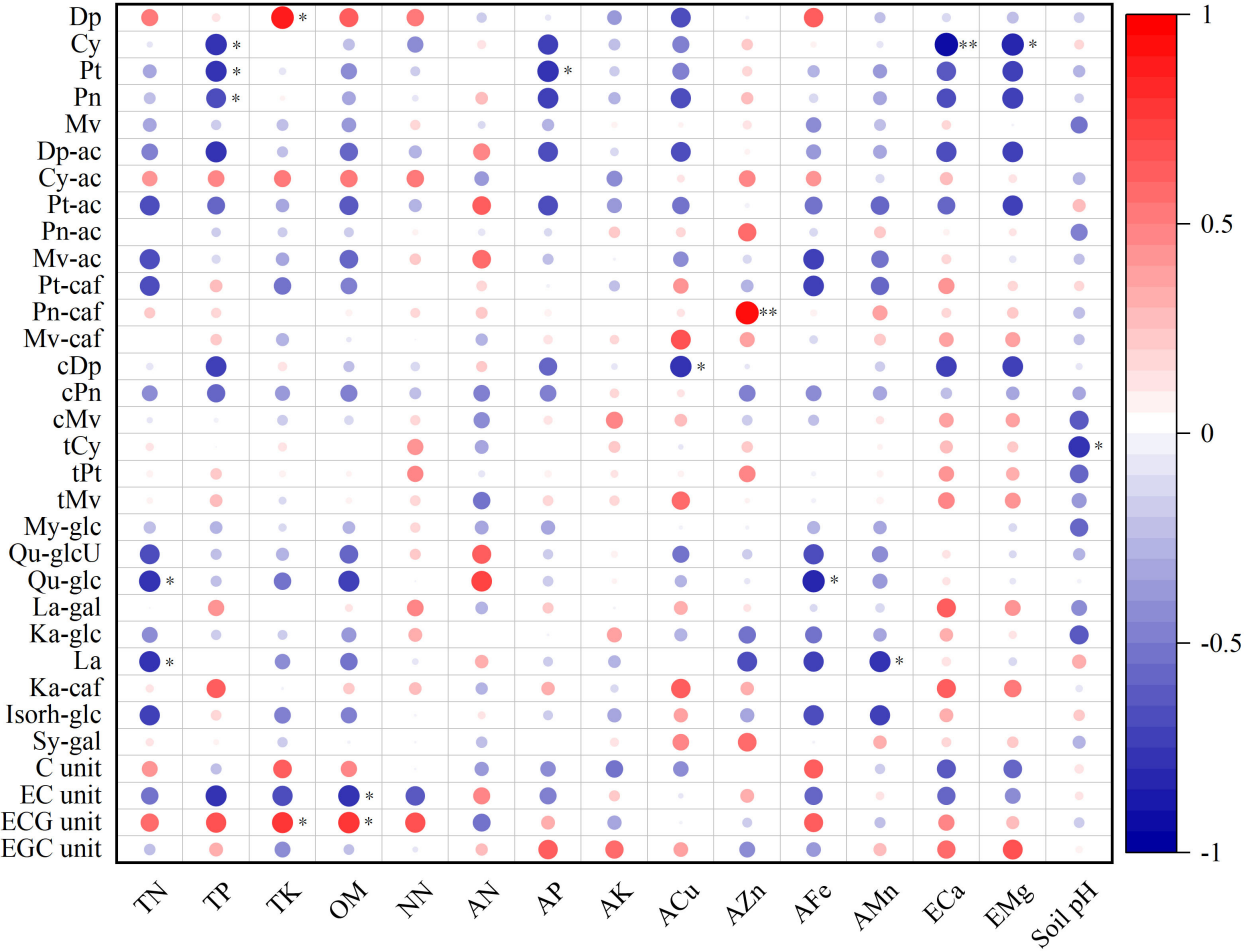
Correlation analysis between soil nutrients and berry flavonoids. TN, TK, TP, OM, NN, AN, AP, AK, ACu, AZn, AFe, AMn, ECa, and EMg are abbreviations for total nitrogen, total potassium, total phosphorus, organic matter, nitrate nitrogen, ammonium nitrogen, available phosphorus, available potassium, available copper, available zinc, available iron, available manganese, exchangeable calcium, and exchangeable magnesium. * and ** indicated that the significance level reached *P* < 0.05 and *P* < 0.01, respectively.

In addition to nitrogen, phosphorus, and potassium, grapevines are highly in demand for calcium and magnesium. The optimal levels of exchangeable calcium were found to be 500–700 mg·kg^−1^ (Wang, 2016), which all the included vineyards did not meet. Therefore, timely calcium supplementation is especially necessary. According to the research results of Wang et al. (2019), the optimal concentration of calcium fertilizer was determined to be 30 kg·ha^-1^ in the vineyard of the eastern foothills of the Helan Mountains. Magnesium plays a crucial role as an element constituent in chlorophyll molecules that regulate photosynthesis processes (Abo El-Ezz et al., 2022). Other studies have indicated that magnesium treatment can inhibit the degradation of anthocyanins and alter the proportion of different anthocyanin monomers (Sinilal et al., 2011). In this study’s results, a significant correlation between magnesium and different anthocyanin monomers was observed (**Figure 6**), indicating that varying magnesium content would have distinct effects on different monomers, ultimately impacting the final color of the fruit.

The presence of trace elements does indeed exert an influence on the composition of wine grapes (Mackenzie and Christy, 2005). The findings of this study corroborate this assertion. Notably, a significant correlation was observed between iron and manganese levels and certain indicators of berry quality (**Figure 3**). Furthermore, it was determined that iron and manganese play pivotal roles in influencing various aspects of fruit quality (**Table 7**). However, based on the classification criteria for soil nutrients in vineyards (Wang, 2016), it was found that the vineyards located in the eastern foothills of the Helan Mountains exhibited a deficiency in iron content. This deficiency may be attributed to high lime content and pH levels within the soil (Dell’Orto et al., 2000). It is important to note that insufficient iron levels can lead to leaf chlorosis and stunted growth in grapevines, ultimately impacting grape berry quality (Bertamini and Neduchezhian, 2005). Therefore, it is imperative to adequately replenish these nutrients in order to regulate grapevine growth and enhance berry quality (Wang et al., 2019; Jiang et al., 2022; Ma et al., 2022).

Soil organic matter plays a crucial role in supplying plant nutrients, improving soil fertility and buffering capacity, as well as enhancing soil physical properties (Fageria, 2012; Waibel et al., 2023). Several studies have indicated a positive relationship between soil organic matter content and soluble solids and reduced sugar contents (Wang, 2016; Li Y. S. et al., 2024), while others have shown a negative correlation between soil organic matter content and soluble solid content (Xu et al., 2013; Qi et al., 2019). In this study, a significant negative correlation was observed between soil organic matter and soluble solids as well as reduced sugar contents, which aligns with the findings of previous research (Qi et al., 2019). This may be attributed to the increasing berry volume associated with higher organic matter levels, resulting in lower soluble solids and reduced sugar contents. Similar trends have been reported in apple and orange production (Liu et al., 2023; An et al., 2024). Research has suggested that the optimal organic matter content falls within the range of 1 – 3% (Aguirre et al., 2023), yet most vineyards in this region do not meet this standard.

The analysis of the correlation between soil nutrient indicators and monomers of the three main flavonoids in grape berries provides a more comprehensive research perspective. Previous studies have primarily focused on the relationship between soil properties and total anthocyanins, total flavonols, and total tannins (Zerihun et al., 2015; Qi et al., 2019; Barbagallo et al., 2021; Wang R. et al., 2021). However, limited research has been conducted on the relationship between soil properties and the individual monomers. This is partly due to the technical challenges associated with detecting monomers, as well as the complexity of data analysis. The flavonoids examined in this study share a common upstream synthesis pathway (phenylpropanoid pathway) (Gouot et al., 2019), resulting in complex internal connections among them. It is worth noting that there have been studies exploring the relationship between soil characteristics and genes related to anthocyanin synthesis, offering insights into understanding how soil nutrients affect anthocyanin monomers (Jiang et al., 2024). This approach can serve as a reference for future analyses of the relationship between soil properties and flavonol and tannin monomers.

Of course, there are certain limitations in this study. Firstly, it is important to note that the quality of wine grape berries is influenced by a combination of factors including local climate, soil composition, vineyard management techniques, and other terroir elements (Meinert, 2018; Qiao et al., 2023). Therefore, while soil nutrients play a role in grape berry quality, they are just one part of the equation. A comprehensive understanding of grape berry quality requires consideration of climatic conditions, management practices, and other contributing factors. Secondly, this study specifically focused on analyzing the relationship between soil nutrient indicators and fruit quality indicators without addressing additional soil characteristics such as physical, chemical and microbial properties. However, it is essential to recognize that these properties and their interactions can impact the availability of soil nutrients. As such, future studies should explore these aspects further. Thirdly, given that both soil nutrients and fruit quality can vary from year to year, it is necessary to conduct continuous studies over multiple years at fixed points in order to verify the stability and reliability of results. Finally, the uptake of soil nutrients by grapevines largely depends on water transport. How to effectively couple water and fertilizer, and enhance their utilization efficiency for different types of soils, is an important scientific question that warrants further exploration by researchers.

## 5 Conclusions

1. The soil of the vineyards in the eastern foothills of the Helan Mountains was alkaline, and most vineyards had poor soil nutrients. With the exception of the DWK vineyard, total nitrogen, total phosphorus and organic matter replenishment were required in all vineyards. In addition, all vineyards required increased application of nitrate nitrogen, ammonium nitrogen, available phosphorus, available iron, available calcium, and available magnesium, as well as reduction of the soil alkalinity. For the vineyard of DWK, ZBB, and YQY, it was needed to reduce the weight and volume of the berries so as to increase in the specific surface area of the berries, which promotes the accumulation of flavor substances in the berries and, thus, the quality of the berries.
2. Multilinearity and strong coordination were observed in the soil nutrient indicators, and there was a close correlation between the soil nutrient indicators and the berry quality indicators. Ammonium nitrogen, available potassium, available copper, available zinc, available iron, and available manganese were the key soil nutrient indicators that affected the highest number of grape berry quality indicators.
3. ZBB and YQY vineyards showed better fruit quality, while HD vineyards showed worse fruit quality. HD vineyards should determine earlier harvest time to reduce the reducing sugar content of the berry, increase the titratable acid content of the berry, and improve the balance of the wine’s mouthfeel.
4. In this study, the internal relationship between soil nutrients and fruit quality was investigated using quantitative methods. The fruit quality of different vineyards was evaluated and ranked. The findings not only provide guidance for vineyard managers to enhance fruit quality through soil improvement, but are also of significant importance in exploring distinct vineyard terroir characteristics and developing differentiated products. In addition, the study provides new insights and ideas for a comprehensive assessment of grape beery quality, efficient fertilizer utilization, classification criteria for wine-producing sub-regions, and contributes to the development of refined vineyard management schemes.

## Data availability statement

The raw data supporting the conclusions of this article will be made available by the authors, without undue reservation.

## Author contributions

YL: Investigation, Software, Writing – original draft, Writing – review & editing. QL: Investigation, Software, Writing – original draft. YY: Methodology, Writing – review & editing. WL: Data curation, Writing – original draft. CX: Conceptualization, Investigation, Writing – original draft. YW: Formal analysis, Methodology, Writing – original draft. LN: Funding acquisition, Investigation, Writing – original draft. XL: Funding acquisition, Supervision, Writing – review & editing.
