# Supplementary material for: Evaluation of soil nutrients and berry quality characteristics of Cabernet Gernischet (Vitis vinifera L.) vineyards in the eastern foothills of the Helan Mountains, China
Source: Front Plant Sci. 2024 Jul 25;15:1418197. doi: 10.3389/fpls.2024.1418197 (PMC11306049; doi:10.3389/fpls.2024.1418197)
Supplement: Supplementary file 1 [file Table_1.docx]

Supplementary Material

# Supplementary Data

## Tables

Supplementary Table 1. The basic physicochemical indicators of wine from Cabernet Gernischet vineyards

| Vineyard | Alcohol (Vol %) | Reducing sugar/(g·L^-1^) | Titratable acidity/(g·L^-1^) | Malic acid /(g·L^-1^) | Lactic acid /(g·L^-1^) | Tartaric acid /(g·L^-1^) | pH | Volatile acid /(g·L^-1^) | Total polyphenol /(g·L^-1^) |
| --- | --- | --- | --- | --- | --- | --- | --- | --- | --- |
| DWK | 11.11±0.04f | 1.07±0.15e | 4.94±0.01e | 1.40±0.03ab | 0.86±0.05b | 0.83±0.03f | 3.87±0.00a | 0.36±0.03c | 1.02±0.02e |
| ZBB | 12.44±0.08d | 1.13±0.12e | 5.40±0.02b | 1.53±0.10a | 0.77±0.06bc | 1.55±0.01b | 3.77±0.02c | 0.29±0.04d | 1.33±0.09cd |
| YQY | 13.53±0.12b | 1.77±0.15c | 4.70±0.02f | 0.83±0.03d | 1.23±0.03a | 1.41±0.01c | 3.87±0.01a | 0.35±0.01c | 1.77±0.01a |
| HYT | 12.63±0.11d | 1.43±0.12d | 4.32±0.02g | 0.46±0.03e | 1.17±0.03a | 1.13±0.01e | 3.81±0.01b | 0.32±0.01cd | 1.27±0.02d |
| SX | 12.24±0.09e | 1.63±0.15cd | 6.03±0.01a | 1.13±0.05c | 0.25±0.05e | 1.25±0.02d | 3.59±0.01e | 0.58±0.01a | 1.08±0.01e |
| BHZ | 12.85±0.02c | 2.13±0.06b | 5.13±0.02d | 0.77±0.02d | 0.67±0.01d | 0.84±0.02f | 3.83±0.01b | 0.51±0.03b | 1.49±0.02b |
| HD | 14.15±0.10a | 2.60±0.10a | 5.24±0.02c | 1.27±0.03bc | 0.68±0.02cd | 1.75±0.01a | 3.70±0.01d | 0.33±0.01cd | 1.43±0.02bc |

Note: Different small letters in the same column mean a significant difference at *P* < 0.05 among vineyards according to Tukey’s test.

Supplementary Table 2. The sensory evaluation of wine from Cabernet Gernischet vineyards

| Vineyard | Appearance | | | | Flavor and aroma | | | Mouthfeel | | | | | | | Total score | Overall rating |
| --- | --- | --- | --- | --- | --- | --- | --- | --- | --- | --- | --- | --- | --- | --- | --- | --- |
|  | Clarity | Brightness | Hue | Chroma | Purity | Intensity | Elegance | Sweetness | Acidity | Bitter | Astringency | Alcohol | Harmony | Aftertaste |  |  |
| DWK | 7.46±0.78a | 7.15±0.90a | 2.69±1.18a | 7.15±1.57a | 6.85±0.90a | 6.46±1.20a | 6.62±1.33a | 4.38±2.66a | 5.46±1.94a | 4.00±2.00a | 5.23±1.74a | 5.08±1.80a | 6.23±1.24a | 5.00±1.78a | 79.77 | good |
| ZBB | 7.38±0.77a | 7.31±1.03a | 2.08±1.12a | 6.92±1.50a | 6.69±1.11a | 6.77±1.36a | 6.77±1.42a | 4.15±2.85a | 5.85±2.08a | 4.46±1.98a | 6.15±1.41a | 5.00±1.78a | 6.00±1.35a | 5.85±1.82a | 81.38 | excellent |
| YQY | 7.46±0.78a | 7.08±0.86a | 1.69±1.03a | 7.15±1.63a | 6.46±1.39a | 6.54±1.39a | 6.77±1.48a | 4.00±2.58a | 6.15±1.82a | 4.85±2.08a | 6.54±0.88a | 5.46±1.66a | 6.38±1.26a | 5.92±1.55a | 82.46 | excellent |
| HYT | 7.770.73a | 7.38±1.04a | 2.08±1.26a | 7.23±1.17a | 5.08±1.93ab | 5.54±1.76a | 4.77±2.05ab | 3.08±1.93a | 5.62±2.10a | 4.62±2.06a | 5.31±2.06a | 5.69±1.70a | 4.85±1.82ab | 5.00±1.73a | 74.00 | good |
| SX | 7.54±0.88a | 7.31±1.11a | 2.38±1.04a | 7.00±1.68a | 5.85±1.99ab | 5.85±1.99a | 5.31±2.36ab | 3.69±2.43a | 5.46±1.61a | 5.08±2.06a | 5.85±1.46a | 4.92±1.89a | 5.46±1.81ab | 5.08±1.44a | 76.77 | good |
| BHZ | 7.54±0.78a | 7.15±0.69a | 2.54±1.13a | 7.08±1.50a | 5.92±1.80ab | 6.23±1.96a | 5.62±2.22ab | 3.85±2.54a | 5.38±1.85a | 4.46±1.94a | 5.08±1.55a | 4.77±1.59a | 5.31±1.65ab | 5.08±1.61a | 76.00 | good |
| HD | 7.38±0.77a | 7.23±0.83a | 2.77±1.36a | 6.85±1.41a | 4.23±1.96b | 5.92±2.06a | 3.92±2.14b | 2.92±1.93a | 5.38±2.29a | 4.46±2.03a | 4.62±2.06a | 5.00±1.96a | 3.77±1.69b | 4.54±1.90a | 69.00 | acceptable |

Note: Different small letters in the same column mean a significant difference at *P* < 0.05 among vineyards according to Tukey’s test; Overall score of 80 or greater is considered excellent, greater than 70 (inclusive) but less than 80 is considered good, greater than 60 (inclusive) but less than 70 is considered acceptable, below 60 is considered unqualified.
